# Enhancement of thermoelectric properties over a wide temperature range by lattice disorder and chemical potential tuning in a (CuI)_*y*_(Bi_2_Te_3_)_0.95−*x*_(Bi_2_Se_3_)_*x*_(Bi_2_S_3_)_0.05_ quaternary system

**DOI:** 10.1039/c8ra09280j

**Published:** 2019-01-31

**Authors:** Hyunyong Cho, Song Yi Back, Jin Hee Kim, Omkaram Inturu, Ho Seong Lee, Jong-Soo Rhyee

**Affiliations:** Department of Applied Physics and Institute of Natural Sciences, Kyung Hee University Yongin 17104 Korea jsrhyee@khu.ac.kr +82-31-204-8122 +82-31-201-2415; Center for Integrated Nanostructure Physics (CINAP), Institute for Basic Science (IBS), Sungkyunkwan University Suwon 16419 Korea; School of Materials Science and Engineering, Kyungpook National University Daegu 41566 Korea

## Abstract

Bi_2_Te_3_-based compounds have received attention as thermoelectric materials for room-temperature cooling and waste heat recovery applications. With potential application prospects, quaternary compounds of Bi_2_Te_3_–Bi_2_Se_3_–Bi_2_S_3_ composites can be used for mid-temperature power generation under 500 °C. Herein, we investigated the thermoelectric properties of (CuI)_*y*_(Bi_2_Te_3_)_0.95−*x*_(Bi_2_Se_3_)_*x*_(Bi_2_S_3_)_0.05_ (*x* = 0.05, 0.2; *y* = 0.0, 0.003) compounds. Through X-ray diffraction and transmission electron microscopy, we confirmed that the lattice disorder in (Bi_2_Te_3_)_0.95−*x*_(Bi_2_Se_3_)_*x*_(Bi_2_S_3_)_0.05_ (*x* = 0.2) was due to multiple element substitutions. Disorder carrier scattering induced the localized nature of electrical resistivity, as confirmed by variable range hopping at low temperature. The temperature-dependent Seebeck coefficient of (Bi_2_Te_3_)_0.95−*x*_(Bi_2_Se_3_)_*x*_(Bi_2_S_3_)_0.05_ showed a carrier-type change from p- to n-type behaviour in the intermediate temperature range (525 K for *x* = 0.05 and 360 K for *x* = 0.2). Even though strong carrier localization increased electrical resistivity, resulting in degradation of the power factor and thermoelectric performance, when the chemical potential was increased to the conduction band minimum through CuI co-doping into the (CuI)_0.003_(Bi_2_Te_3_)_0.95−*x*_(Bi_2_Se_3_)_*x*_(Bi_2_S_3_)_0.05_ (*x* = 0.05, 0.2) compounds, the carriers were delocalized and showed n-type behaviour in the Seebeck coefficient. The temperature-dependent thermal conductivity shows the suppression of bipolar conduction behaviour. The simultaneous effect on carrier optimization through chemical potential tuning and lattice disorder caused a high *ZT* value of 0.85 at 523 K for CuI-doped (Bi_2_Te_3_)_0.75_(Bi_2_Se_3_)_0.2_(Bi_2_S_3_)_0.05_, which was comparatively high for n-type thermoelectric materials in the mid-temperature range.

## Abstract 2

Temperature-dependent *ZT* values of (CuI)_*y*_(Bi_2_Te_3_)_0.95−*x*_(Bi_2_Se_3_)_*x*_(Bi_2_S_3_)_0.05_ (*x* = 0.05, 0.2; *y* = 0.0, 0.003) compounds compared with other related n-type compounds.

## Graphical abstract

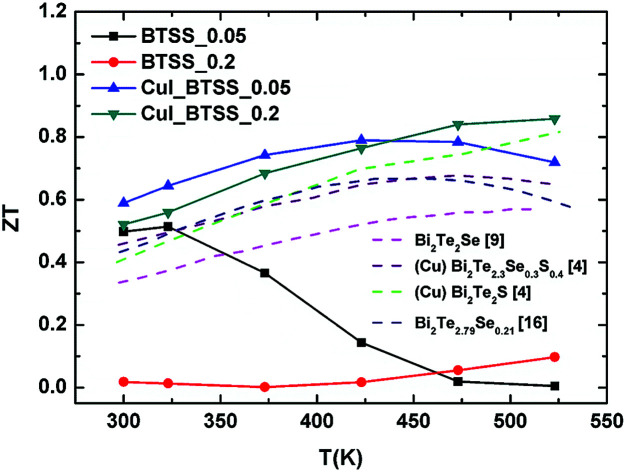

## 1. Introduction

High-performance thermoelectric (TE) materials that can directly and reversibly convert heat and electrical energy are widely utilized in various devices, such as heat power generators or thermoelectric coolers. The performance of an thermoelectric material is defined by its dimensionless figure-of-merit (*ZT* = *S*^2^*σT*/*κ*), where *S*, *σ*, *κ*, and *T* are the Seebeck coefficient, electrical conductivity, thermal conductivity, and absolute temperature, respectively.^1,2^

Bismuth telluride-based compounds are good TE materials at room temperature and mainly used in solid state refrigeration technologies. Furthermore, high thermoelectric performance has been reported for the compositional change of Bi_2_Te_3_-based materials up to the mid-temperature region.^3,4^ Bismuth telluride-based materials are increasingly interesting for low-temperature power applications.^5–14^ For example, Wang *et al.* reported that a high *ZT* of ∼0.86 at 600 K was reached by n-type Bi_2_Te_1.5_Se_1.5_ with I-doping.^15^ Tang *et al.*^5^ reported that the maximum *ZT* value of 0.1 at% SbI–Bi_2_Te_1.9_Se_1.1_ alloy was 1.1 at 600 K due to the band engineering and hot-deformation process.^5^ Indeed, n-type Bi_2_Te_2_S/Se showed a peak *ZT* of ∼0.8 at 573 K owing to the alloying effect.^4^ The inherent properties of Bi–Te-based materials include many disorders, such as dislocations, distortions, anti-site defects, and point defects, from each synthesis and sintering process.^9,16,17^ Anti-site defects (Bi_Te_, Te_Bi_) and Te-vacancies in the Bi–Te matrix generate holes and electrons, respectively. Furthermore, a reduction in thermal conductivity is expected due to the formation of dislocations, point defects, and lattice distortions.^18^ However, for inorganic thermoelectric materials systems, there have been few studies on how carrier transport is affected by an induced disorder system. Variable range hopping (VRH) is caused by random disorder, which has a random potential in the matrix. Furthermore, owing to the hopping carrier transport, conductive carriers in the matrix are localized by random potential. VRH can also be confirmed at low temperatures by electrical transport measurements, while acoustic and optical phonon scattering of carriers can be neglected in the transport mechanism.^19,20^ Herein, we investigated the thermoelectric properties of (CuI)_*y*_(Bi_2_Te_3_)_0.95−*x*_(Bi_2_Se_3_)_*x*_(Bi_2_S_3_)_0.05_ (*x* = 0.05, 0.2; *y* = 0.0, 0.003) compounds. To induce many lattice disorders, we attempted lattice mismatch with the main matrix (Bi_2_Te_3_) by doping with selenium and sulfur as substitutes for tellurium. Especially, in (Bi_2_Te_3_)_0.95−*x*_(Bi_2_Se_3_)_*x*_(Bi_2_S_3_)_0.05_ (*x* = 0.2), peak broadening of the X-ray diffraction (XRD) pattern was caused by lattice distortion or lattice strain in comparison with (Bi_2_Te_3_)_0.95−*x*_(Bi_2_Se_3_)_*x*_(Bi_2_S_3_)_0.05_ (*x* = 0.05). Furthermore, weak superstructure peaks due to lattice distortion were confirmed using electron diffraction patterns. Regarding the electrical resistivity of (Bi_2_Te_3_)_0.95−*x*_(Bi_2_Se_3_)_*x*_(Bi_2_S_3_)_0.05_ (*x* = 0.05.0.2), VRH appeared due to disorder carrier scattering at low temperature. Owing to carrier localization, carrier hopping had a negative effect on the thermoelectric properties. To enhance thermoelectric performance, we attempted to shift the chemical potential to a delocalized state using Cu/I co-doping. Furthermore, CuI-doped compounds reduced the bipolar effect at high temperatures. Consequently, a *ZT* of 0.85 was achieved with n-type materials at 523 K using CuI-doped (Bi_2_Te_3_)_0.75_(Bi_2_Se_3_)_0.2_(Bi_2_S_3_)_0.05_, which is a moderately high *ZT* value for n-type materials at a mild temperature for waste heat power generation. From a novel perspective, we have demonstrated the effect of induced disorder on the carrier transport region in the matrix and confirmed that thermoelectric performance was increased by chemical potential tuning, even in the presence of disorder.

## 2. Experimental

Elemental Bi (99.999%), Te (99.999%), Se (99.999%), S (99.999%), and CuI (99.99%) were weighed to obtain stoichiometry of (CuI)_*y*_(Bi_2_Te_3_)_0.95−*x*_(Bi_2_Se_3_)_*x*_(Bi_2_S_3_)_0.05_ (*x* = 0.05, 0.2; *y* = 0.0, 0.003) and then loaded into a quartz ampoule. The ampoule was sealed under a high vacuum, melted at 1073 K for 24 h, and then cooled slowly for 30 h in the furnace. The product was then pulverized by hand grinding in a mortar under a pure argon atmosphere. The resulting powder was sintered by hot-pressing in a graphite die (diameter, 12.7 mm) at 773 K for 1 h under a uniaxial pressure of 50 MPa.

Phase identification and structural characterization of all the bulk samples were performed by X-ray diffraction (XRD) using Cu Kα radiation. High-resolution transmission electron microscopy (HRTEM) was used to characterize the microstructure of samples prepared using a focused ion beam (FIB).

The thermoelectric properties of all samples were measured along the in-plane direction. The Seebeck coefficient and electrical conductivity were measured by a four-probe method using a commercial ZEM-3 (ULVAC-RIKO, Japan) system. The thermal conductivity was obtained using the equation *κ* = *ρ*_s_*λC*_p_, where *ρ*_s_, *λ*, and *C*_p_ are the sample density, thermal diffusivity, and specific heat, respectively. The thermal diffusivity *λ* was measured using the laser flash method (LFA-457, Netzsch, Germany) and the specific heat (*C*_p_) was measured, with high temperature extrapolation obtained from the data using a physical property measurement system (PPMS Dynacool 14 T, Quantum Design, U.S.A.). The Hall resistivity (*ρ*_*xy*_) and electrical resistivity (*ρ*) below 300 K were measured by the four-probe contact method using PPMS. The Hall carrier concentrations (*n*_H_) were obtained using the equation *n*_H_ = −1/(*R*_H_*e*), where *R*_H_ = *ρ*_*xy*_/*H*, *e*, and *H* are the Hall coefficient, electron charge, and applied magnetic fields, respectively.

## 3. Results and discussion

### 3.1 Experimental characterization

**Fig. 1:**
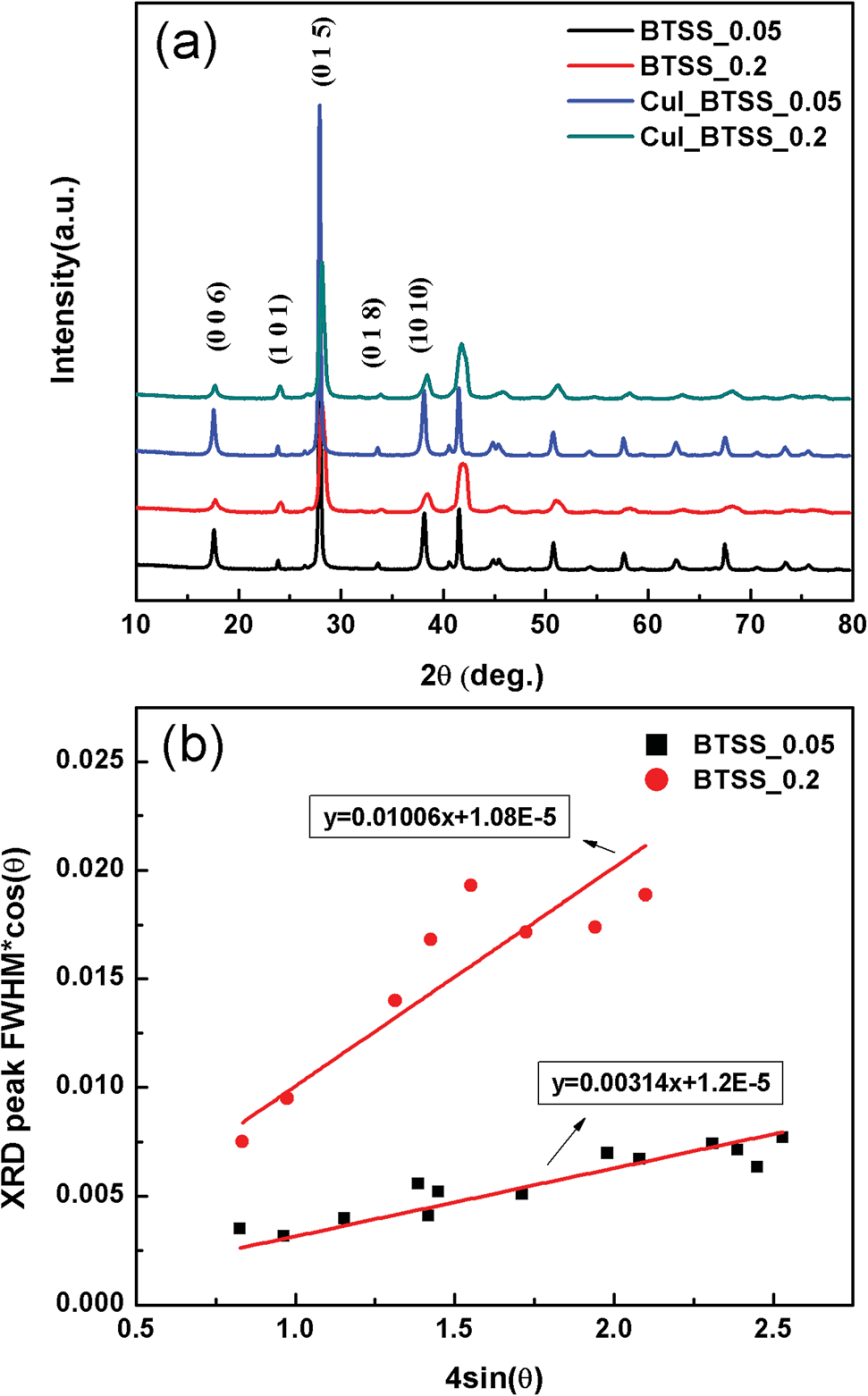
(a) Powder X-ray diffraction patterns of (CuI)_*y*_(Bi_2_Te_3_)_0.95−*x*_(Bi_2_Se_3_)_*x*_(Bi_2_S_3_)_0.05_ (*x* = 0.05, 0.2; *y* = 0.0, 0.003) bulk samples. (b) Angle-dependent XRD peak widths, according to the Williamson–Hall relation.

Fig. 1(a) shows X-ray diffraction (XRD) patterns of the (CuI)_*y*_(Bi_2_Te_3_)_0.95−*x*_(Bi_2_Se_3_)_*x*_(Bi_2_S_3_)_0.05_ (CuI_BTSS) (*x* = 0.05, 0.2; *y* = 0.0, 0.003) polycrystalline compounds, indicating that the major diffraction peaks were well indexed to the theoretical rhombohedral peaks of Bi_2_Te_2_Se (space group no. 166).

Variation of the Se concentration and CuI doping shifted the diffraction peaks to higher angles. The lattice parameters of (CuI)_*y*_(Bi_2_Te_3_)_0.95−*x*_(Bi_2_Se_3_)_*x*_(Bi_2_S_3_)_0.05_ (*x* = 0.05, 0.2; *y* = 0.0, 0.003) are listed in Table 1.

**Table 1:** Lattice parameters of (CuI)_*y*_(Bi_2_Te_3_)_0.95−*x*_(Bi_2_Se_3_)_*x*_(Bi_2_S_3_)_0.05_ (*x* = 0.05, 0.2; *y* = 0.0, 0.003) compounds

Lattice parameter	*a* (Å)	*c* (Å)
BTSS_0.05	4.3446	30.2473
BTSS_0.2	4.3023	30.0629
CuI_BTSS_0.05	4.3483	30.2638
CuI_BTSS_0.2	4.3033	30.0898

The lattice parameters of (Bi_2_Te_3_)_0.95−*x*_(Bi_2_Se_3_)_*x*_(Bi_2_S_3_)_0.05_ (*x* = 0.05, 0.2) (BTSS_0.05, BTSS_0.2) decreased in the *a*- and *c*-axis with increasing Se concentration. When CuI was doped into BTSS, the lattice parameters of the *a*- and *c*-axis were slightly increased, probably due to Cu atoms located in the van der Waals gap and iodine substitution into the Te vacancy site.^21,22^ In the XRD patterns, peak broadening with increasing 2*θ* value was observed in samples of BTSS_0.2 and CuI-doped BTSS_0.2 compared with those of BTSS_0.05 and CuI-doped BTSS_0.2 with increasing Se concentration.

In general, XRD peak broadening was caused by crystal domain size and micro-strain. The Williamson–Hall relation represents the lattice strain and grain size from lower resolution XRD data from peak position and peak widths according to the following equation:^23–25^1
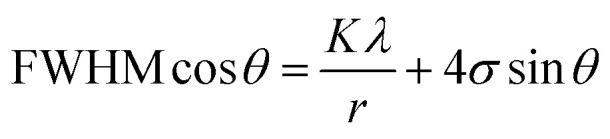
where *r* is the crystalline domain size, *σ* is the micro-strain fluctuation, *λ* is the incident X-ray wavelength, and *K* is a dimensionless shape factor with a typical value of 0.9. This equation shows a convenient correlation between the full width at half maximum (FWHM) and diffraction angle. If the grain size (*r*) is large enough, peak broadening of the XRD pattern is predominantly due to micro-strain, which is caused by non-uniform lattice distortion, dislocation, anti-phase domain boundaries, and grain surface relaxation, among other factors. According to representative data in Fig. 1(b), both samples (BTSS_0.05, 0.2) had y-intercepts close to zero, which indicated large crystalline domain sizes. Furthermore, with increasing Se concentration, the BTSS_0.2 slope increased about three-fold compared with that of BTSS_0.05, which indicated the increased lattice strain in BTSS_0.2. For Cu/I co-doped samples, we expected to see a similar trend to non-doped samples owing to the extent of peak broadening being maintained. As a result, through Se and S doping, we confirmed that disorder was induced into the matrix due to lattice mismatch.

**Fig. 2:**
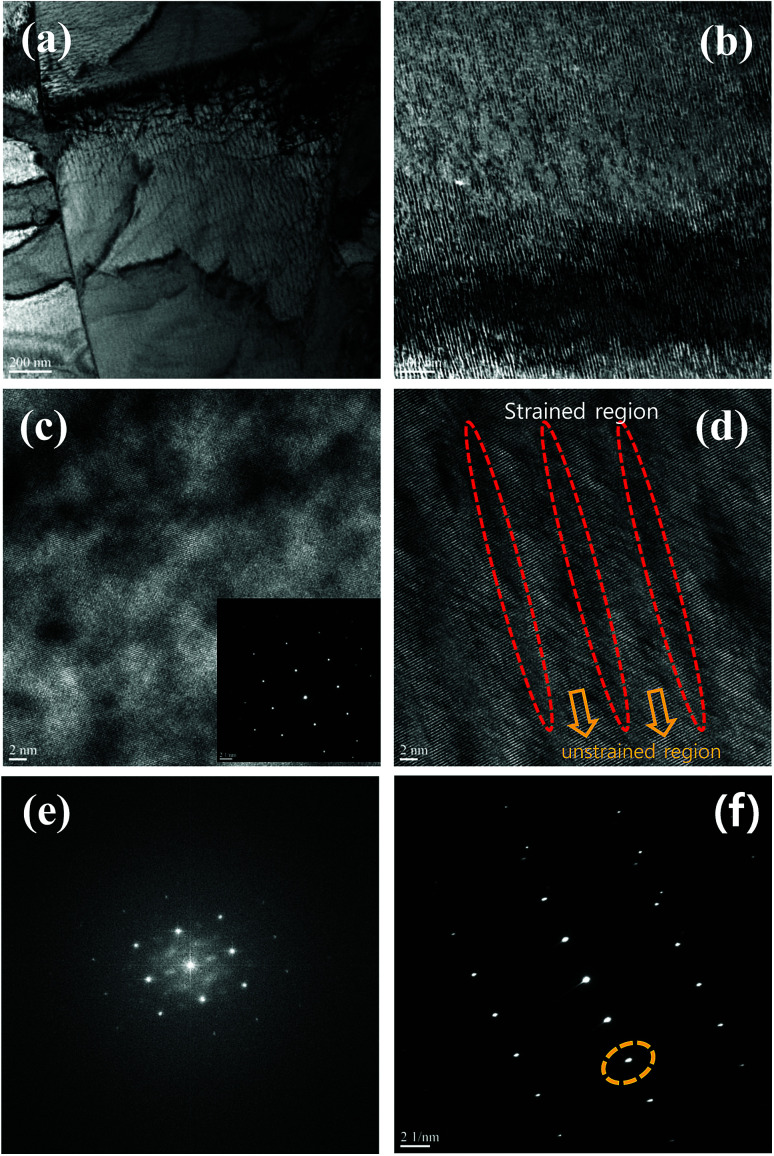
Microstructures of (Bi_2_Te_3_)_0.95−*x*_(Bi_2_Se_3_)_*x*_(Bi_2_S_3_)_0.05_ (*x* = 0.05, 0.2) (BTSS_0.05 and BTSS_0.2): (a) bright field micrograph of BTSS_0.05, (b) bright field micrograph of BTSS_0.2, (c) HR-TEM image of BTSS_0.05 and its electron diffraction (ED) pattern (inset), (d) HR-TEM image of BTSS_0.2, and (f) ED pattern of BTSS_0.2, (e) ED pattern of low-magnification TEM image of BTSS_0.05.

Interestingly, BTSS_0.05, 0.2 samples had a high density of nanoscale distorted regions, as shown in Fig. 2.

The high-resolution transmission electron microscopy (HR-TEM) image in Fig. 2(d) (BTSS_0.2 sample) shows that the strain and unstrain regions (or the strain field domains) were clearly separated (marked with red ellipses) from lattice distortion. The fast Fourier transform (FFT) electron diffraction image (Fig. 2(f)) shows super lattice peaks induced by the lattice strain field. Furthermore, the elongated spot (marked with yellow dashed circles in Fig. 2(f)) was due to some rotation of the unstrained region, indicating that there was strong stress in the matrix. The BTSS_0.05 sample possessed lattice strain, showing a weak spot in the FFT image (Fig. 2(c) and (e)). However, the bright field TEM image in Fig. 2(b) shows that the BTSS_0.2 sample had many bright lines compared with the BTSS_0.05 sample (Fig. 2(a)), which had a relatively weak bright line. Therefore, for the BTSS_0.2 sample, many lines caused by the lattice strain effect were observed with increasing Se concentration compared with the BTSS_0.05 sample, despite the strain effect being present in the matrix of both samples. This confirmed that the XRD peak broadening of BTSS_0.2 sample was predominantly caused by the lattice strain effect in the matrix.

### 3.2 Carrier transport at low temperature

**Fig. 3:**
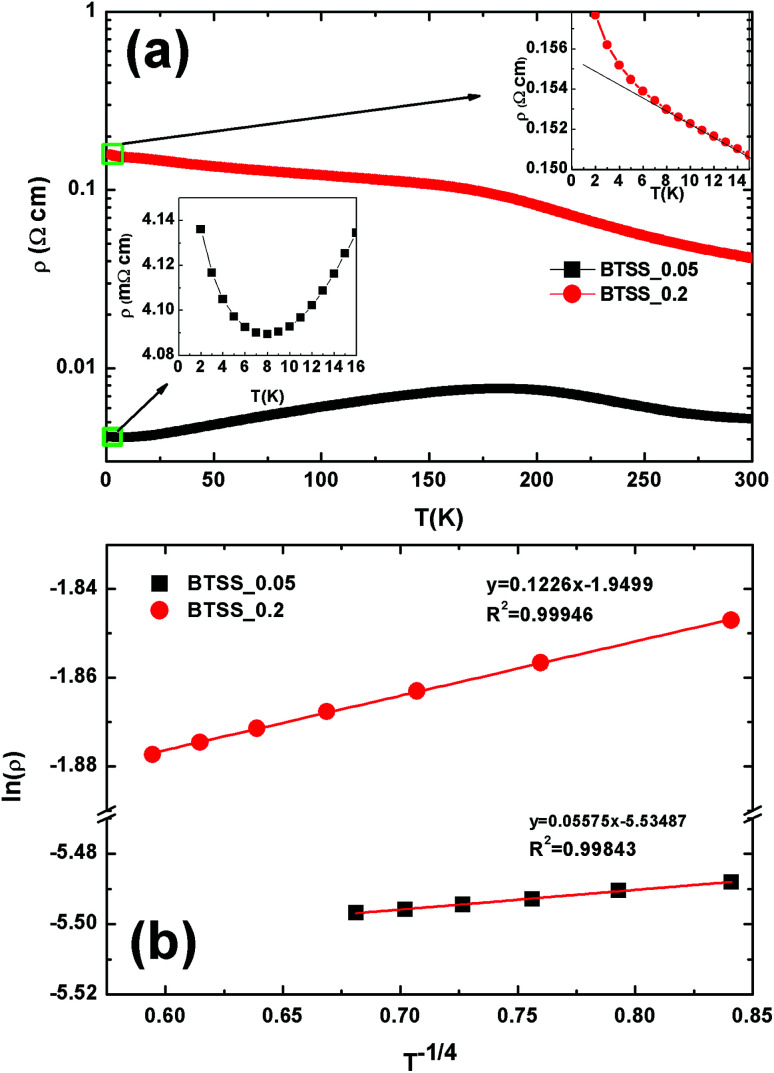
(a) Temperature-dependent electrical resistivity of (Bi_2_Te_3_)_0.95−*x*_(Bi_2_Se_3_)_*x*_(Bi_2_S_3_)_0.05_ (*x* = 0.05, 0.2; BTSS_0.05 and BTSS_0.2) and electrical resistivity at low temperature below 10 K (inset); (b) plots of ln *ρ vs. T*^−1/4^ of BTSS_0.05 and BTSS_0.2 below 10 K.

In materials containing a large number of defects or with a disordered system, the electrons are substantially affected by random potentials. The electrons, which can travel by tunnelling or hopping between nearest sites, are strongly affected by the potential at low temperature, and can be localized by lattice disorder effects, such as variable range hopping (VRH) transport.^19,20,26,27^ To confirm carrier localization at low temperature in the disorder system, we examined the electrical resistivity of all samples measured at low temperature. The temperature-dependent electrical resistivity *ρ*(*T*) of the BTSS_0.05 and BTSS_0.2 poly crystalline compounds is shown in Fig. 3(a).

The BTSS_0.05 and BTSS_0.2 samples showed metallic and semiconducting behaviour, respectively, from ∼200 K to 2 K. However, near 10 K, the slope of electrical resistivity slightly changed with decreasing temperature, as shown in the inset to Fig. 3(a). The universal hopping conductivity is expressed using the following equation:

2 *ρ*(*T*) = *ρ*_0_(*T*)exp(*T*_0_/*T*)^*p*^

where *ρ*(*T*) is the electrical resistivity, *ρ*_0_(*T*) is the pre-exponential factor, *T*_0_ is the characteristic temperature, and *p* is the exponent depending on the hopping mechanism. In Fig. 3(b), we plotted ln *ρ*(*T*) *vs. T*^−*p*^ for BTSS_0.05 and BTSS_0.2 below 8 K. Evidently, our data showed a straight line proportional to *T*^−1/4^, meaning that the hopping mechanism was Mott-type VRH related to the induced disorder system.^19,20,28^ In general, the pre-factor is temperature-dependent *ρ*_0_(*T*), such as *ρ*_0_(*T*) = *AT*^*q*^, where *A* is a constant and *q* is the exponent depending on the regime of the hopping conductivity.^29^ When *p* = 1/4 and *q* = −3/4, we observed that the equation fitted our data well, and *T*_0_ was estimated using the VRH equation. Furthermore, using the obtained carrier concentration in Table 2, the localization length (*ξ*) was calculated using *T*_0_ = 16(1/*k*_B_*N*(*E*_F_)*ξ*^3^), where *k*_B_ is the Boltzmann constant and *N*(*E*_F_) is the density of states at the Fermi surface. The localization lengths of BTSS_0.05 and BTSS_0.2 were about 9.2 nm and 8.7 nm, respectively. This confirmed that the carriers related to the formation of induced disorders were localized by VRH at low temperature in BTSS_0.05 and BTSS_0.2.

**Table 2:** Hall carrier concentration (*n*_H_), Hall mobility (*µ*_H_), and mean free path *l* (MFP) of (CuI)_*y*_(Bi_2_Te_3_)_0.95−*x*_(Bi_2_Se_3_)_*x*_(Bi_2_S_3_)_0.05_ (*x* = 0.05, 0.2; *y* = 0.0, 0.003) compounds

Temperature	*n*_H_ (10^19^ cm^−3^)		*µ*_H_ (cm^2^ V^−1^ S^−1^)		*l* (nm)
	10 K	300 K	10 K	300 K	300 K
BTSS_0.05	0.75	1.1	203	105	4.8
BTSS_0.2	0.25	1.4	17	11	0.54
CuI_BTSS_0.05	4.5	5.8	718	115	9
CuI_BTS_0.2	5.2	5.0	728	188	14

**Fig. 4:**
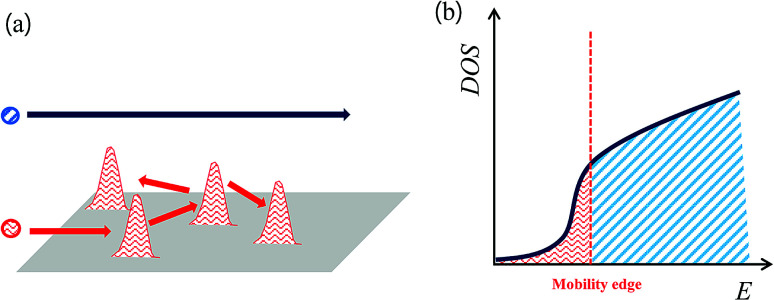
Schematic diagrams of (a) localization of carriers (red arrows) with delocalized carriers (blue arrow), respectively. (b) Density of states (DOS) of disordered system. Mobility edge due to charge localization gives rise to a sharp density of states.

Normally, the density of states (DOS) in crystalline solids is parabola-shaped. However, in a disordered system, exponential tails appear due to some electronic state based on the disorder or defects state in the DOS. Furthermore, the mobility edge, divided into the localization and conductive regions, occurs due to disorder, as shown in Fig. 4(b).^19,27^

If the Fermi level is in the localized region (marked with red zone below the mobility edge), the electrons can only move through hopping or tunnelling mechanisms. In other words, the red arrows in Fig. 4(a) represent localized carriers caused by the disorder potential. Therefore, when carrier localization occurred in the matrix through induced disorder, the electrical conductivity was expected to become insulating behaviour. To overcome poor electrical conductivity, first, the disorder must be reduced,^27^ or second, in the band structure, the chemical potential can be tuned from the localized state to delocalized state through doping without reducing the disorder. Here, we attempted to tune the chemical potential using Cu/I co-doping. The temperature-dependent electrical resistivity *ρ*(*T*) of the CuI-doped BTSS_0.05 and BTSS_0.2 poly crystalline compounds showed that both samples had metallic or highly degenerated semiconducting behaviour in all temperature regions. In particular, the CuI-doped BTSS_0.2 sample showed significantly decreased electrical resistivity compared with the BTSS_0.2 sample. Furthermore, both CuI-doped samples were delocalized owing to the increase in chemical potential to a delocalized region. In Fig. 4(a) and (b), when the chemical potential was located in highly degenerated region, the carriers can move freely (marked with blue arrow and sky-blue region). However, the changes in XRD peak broadening (see Fig. 1(a)) seemed to be negligible in the CuI-doped and un-doped samples, indicating that the disorder was almost unchanged and the chemical potential was moved to overcome carrier localization.

### 3.3 Thermoelectric properties

**Fig. 5:**
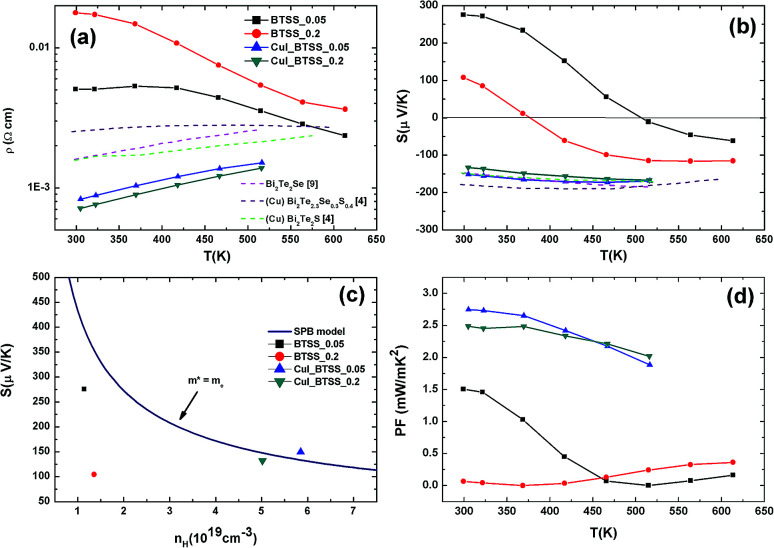
Temperature-dependent electrical properties of (CuI)_*y*_(Bi_2_Te_3_)_0.95−*x*_(Bi_2_Se_3_)_*x*_(Bi_2_S_3_)_0.05_ (*x* = 0.05, 0.2; *y* = 0.0, 0.003) bulk samples: (a) electrical resistivity *ρ*(*T*), (b) Seebeck coefficient *S*(*T*), (c) room-temperature Pisarenko plot of *S vs. n*_H_, and (d) power factor *S*^2^*σ*(*T*) of the (CuI)_*y*_(Bi_2_Te_3_)_0.95−*x*_(Bi_2_Se_3_)_*x*_(Bi_2_S_3_)_0.05_ (*x* = 0.05, 0.2; *y* = 0.0, 0.003) compounds. Pink, purple, green dashed lines represent *ρ*(*T*) (a) and *S*(*T*) (b) for Bi_2_Te_2_Se,^9^ Cu-doped Bi_2_Te_2.3_Se_0.3_S_0.4_,^4^ and Cu-doped Bi_2_Te_2_S,^4^ respectively, from other references.

The temperature-dependent electrical resistivity *ρ*(*T*) of the (CuI)_*y*_(Bi_2_Te_3_)_0.95−*x*_(Bi_2_Se_3_)_*x*_(Bi_2_S_3_)_0.05_ (*x* = 0.05, 0.2; *y* = 0.0, 0.003) polycrystalline compounds is shown in Fig. 5(a).

The *ρ*(*T*) values of the BTSS_0.05 and BTSS_0.2 samples were larger than those of the CuI-doped samples (CuI_0.003__BTSS_0.05 and 0.2) owing to the chemical potential being located in the electrical localized region. Furthermore, with increasing temperature, the *ρ*(*T*) value of un-doped samples decreased because the influence of localization was weakened at high temperatures in both samples. In contrast, the resistivity of all CuI-doped samples showed metallic behaviour with increasing temperature. These results were compared with the *ρ*(*T*) values of Bi_2_Te_2_Se,^9^ Cu-doped Bi_2_Te_2.3_Se_0.3_S_0.4_,^4^ and Cu-doped Bi_2_Te_2_S,^4^ from other references, as represented by pink, purple, and green dashed lines, respectively (Fig. 5). These compounds exhibited highly degenerated semiconducting behaviour, even in the high-temperature region.

The Seebeck coefficients *S*(*T*) of CuI-doped and un-doped samples are shown in Fig. 5(b). Unlike doped samples, which preserved n-type Seebeck coefficients at high temperatures, for un-doped samples with increasing temperature underwent a sign change from p-type to n-type Seebeck coefficients at intermediate temperatures of 350 K (BTSS_0.2) and 525 K (BTSS_0.05). This phenomenon, which is related to impurities in the materials, has occasionally been reported previously.^30–32^ As mentioned earlier, with induced disorder, band tails appear, which results in carrier localization. When the *S*(*T*) values of CuI-doped BTSS_0.05 and 0.2 samples were compared with other reported values (dashed lines),^4,9^ they exhibited relatively high Seebeck coefficients compared with relatively high electrical conductivity, as shown in Fig. 5(a).

Fig. 5(c) shows the Pisarenko plot of *S* with respect to Hall carrier density, *n*_H_. From the Pisarenko relation of each sample, the effective masses related to the gradient of DOS were estimated from the carrier concentration and Seebeck coefficient at 300 K (Table 2). The behaviours of the CuI-doped samples were relatively consistent with the Pisarenko relation (m* = *m*_e_). However, the effective masses of carriers in un-doped samples were lower compared with those of the CuI-doped samples, meaning that the un-doped samples showed non-parabolicity in the DOS or band structure.

Herein, we propose that the small effective mass is due to the chemical potential being located near the band tail, with the non-parabolicity in the DOS occurring due to the band tail formed by induced disorder, as shown in Fig. 4(b) (marked with red zone). Commonly, bismuth–telluride-based materials have native anti-site defects, such that the impurity level or band is positioned between the narrow band gap in the DOS.^33^ When the chemical potential resides near the localized region of the conduction band near the band tail, transferred electrons from the anti-site impurity band are located below the mobility edge state due to thermal energy. Therefore, the holes generated by the impurity band can move freely relative to the localized electrons owing to the disordered potentials. Consequently, in low temperature regions, the holes in un-doped samples can become dominant carriers for electrical transport.

The Hall carrier density (*n*_H_) and Hall mobility (*µ*_H_) values in Table 2 show that CuI doping enhanced the Hall carrier concentration and Hall mobility. In usual cases, Hall carrier concentration is inversely proportional to the Hall mobility owing to electron–electron scattering. This exotic behaviour can be attributed to the carrier localization-to-delocalization transition *via* CuI doping. In pristine BTSS compounds, the disordered lattices localize charge carriers, as confirmed by the variable range hopping transport of carriers at low temperature, resulting in a low carrier concentration and low mobility. In contrast, a small CuI doping increased the chemical potential to the bottom of the conduction band and delocalized carriers, which induced an increased Hall carrier concentration and Hall mobility.

Although the *ρ*(*T*) value of the BTSS_0.2 sample was higher than that of the BTSS_0.05 sample, the *S*(*T*) was a lower value near room temperature. In other words, the BTSS_0.2 sample was strongly localized compared with the BTSS_0.05 sample, while the *S*(*T*) value had a different tendency to electrical resistivity. From the Hall measurement, the calculated carrier concentration in the BTSS_0.2 sample was lower than that of the BTSS_0.05 sample at 10 K, but slightly higher at 300 K, as shown in Table 2. In contrast, the carrier mobility (see Table 2) of the BTSS_0.05 sample was about nine-fold higher than that of the BTSS_0.2 sample, meaning that the localized region in the DOS of the BTSS_0.2 sample was larger than that of BTSS_0.05 sample. Therefore, we suggested that the band tail expanded due to induced disorder in the BTSS_0.2 sample overlapping with the impurity band, such that the *S*(*T*) value was lower than that of the BTSS_0.05 sample owing to hole generation from the impurity band or valence band, even at low thermal energy.

Normally, the variation in carrier concentration represents an opposite trend to carrier mobility within the carrier–carrier scattering mechanism. Interestingly, the carrier density and mobility for both samples were significantly increased by CuI-doping compared with the un-doped samples. In particular, in CuI-doped BTSS_0.2, the carrier mobility and concentration were increased about 17- and 5-fold, respectively, owing to the chemical potential shift to the delocalized region in the DOS. We predict that the carriers freed from localized potential are related to enlargement of the mean free path (MFP) of carriers or weakened electron scattering. The carrier MFP of the samples was estimated using the following equation:^16,34^3
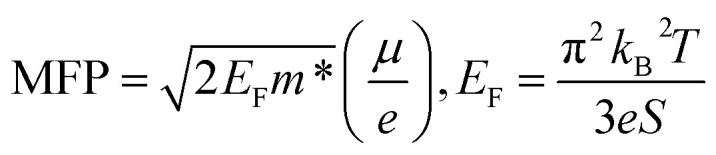
where *E*_F_, m*, *µ*, *k*_B_, and *S* are the Fermi energy, single band effective mass, carrier mobility, Boltzmann constant, and Seebeck coefficient, respectively. Interestingly, in the CuI-doped and undoped BTSS_0.2 samples, the calculated carrier MFPs were 14 nm and 0.54 nm, respectively. Furthermore, the carrier MFP of all doped samples was increased compared with that of the un-doped sample. This confirmed that the carriers of un-doped samples were localized by disorder potentials and released by a chemical potential tuned to the delocalized region.

The temperature-dependent power factor (PF, *S*^2^*σ*) of the samples is shown in Fig. 5(d). The PF values of BTSS_0.05 and BTSS_0.2 samples were relatively low compared with those of the doped samples due to the sign change of *S*(*T*), while the PF values for the CuI-doped samples was significantly enhanced owing to improved electrical transport at room temperature. Accordingly, we confirmed that the thermoelectric properties of CuI-doped samples were improved by chemical potential tuning toward the conduction band side.

**Fig. 6:**
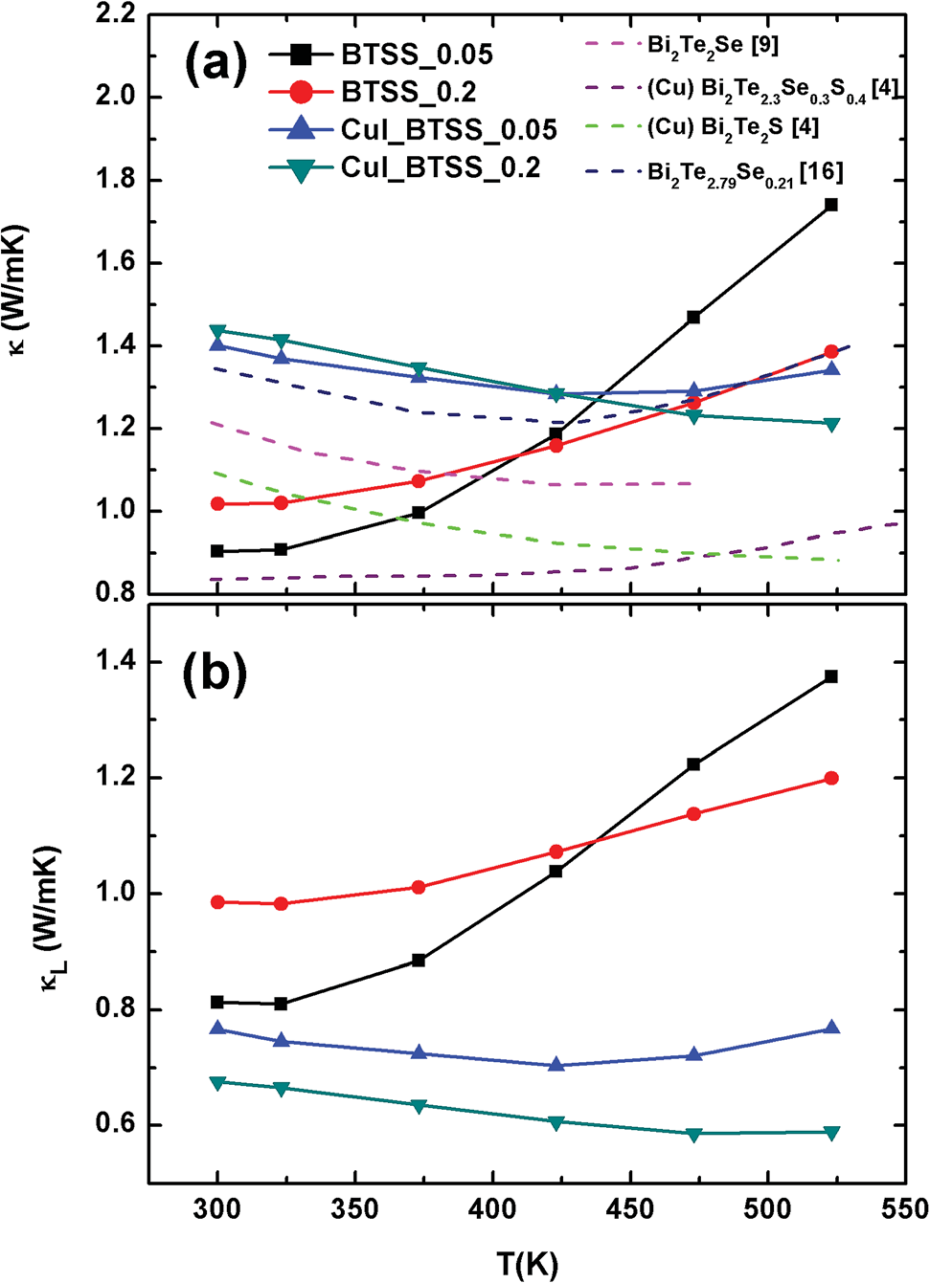
Temperature-dependent (a) total thermal conductivity *κ* and (b) lattice thermal conductivity *κ*_L_ of (CuI)_*y*_(Bi_2_Te_3_)_0.95−*x*_(Bi_2_Se_3_)_*x*_(Bi_2_S_3_)_0.05_ (*x* = 0.05, 0.2; *y* = 0.0, 0.003) compounds. Pink, purple, green, and blue dashed lines represent *κ*(*T*) (a) for Bi_2_Te_2_Se,^9^ Cu-doped Bi_2_Te_2.3_Se_0.3_S_0.4_,^4^ Cu-doped Bi_2_Te_2_S,^4^ and Bi_2_Te_2.79_Se_0.21_ (ref. 16) respectively, from other references.

The total *κ* and lattice thermal conductivity (*κ*_L_) are shown in Fig. 6(a) and (b), respectively. The total thermal conductivity (*κ*) consists of the electronic (*κ*_el_) and lattice (*κ*_lat_) thermal conductivities. *κ*_el_ can be expressed by the Wiedemann–Franz law, *κ*_e_ = *LT*/*ρ*, where *L* is the Lorenz number. In a conventional metallic system, the Lorenz number is given by *L*_0_ = 2.45 × 10^−8^ W Ω K^−2^. However, in correlated metal and degenerated semiconductors, the Lorenz number is not equal to *L*_0_. Therefore, the Lorenz number should be estimated using the following equation within a single parabolic band assumption:^35^4
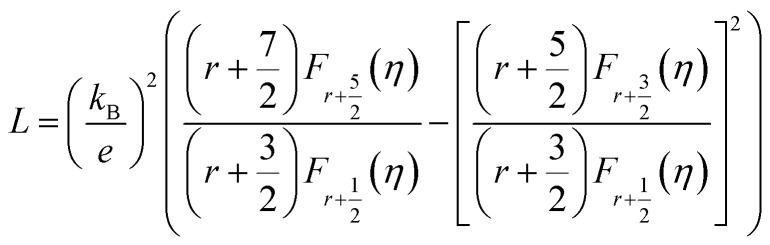
where *η* = *E*_F_/*k*_B_*T* is the reduced Fermi energy, *r* is the scattering parameter, and *F*_*n*_(*η*) is the *n*^th^-order Fermi integral given by5
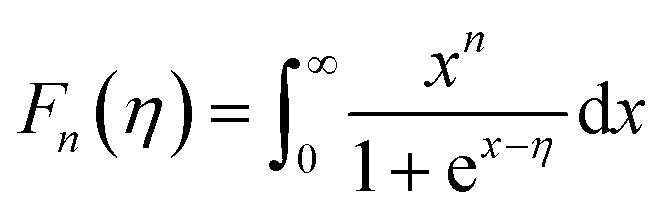


The scattering parameter for phonon scattering is *r* = −1/2. The reduced Fermi level *η* can be obtained from the Seebeck coefficient.6
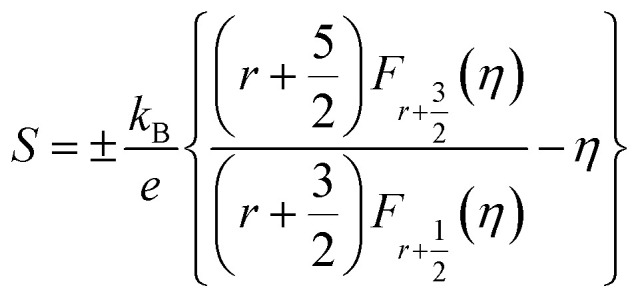


The calculated Lorenz numbers *L* of samples were estimated within the above parabolic band assumption. The calculated *L* values for CuI-doped samples were reasonable in degenerated Bi–Te-based materials. However, it was not clear whether the variation in *L* values for un-doped compounds was large due to the sign change of *S*(*T*). Therefore, the BTSS_0.05 and 0.2 samples deviated from the single parabolic band system. The band tail effect induced by disorder in the DOS is likely related to variation in the *L* value. The temperature-dependent *κ* of the samples is shown in Fig. 6(a). The *κ* value of CuI-doped BTSS_0.05 decreased with increasing temperature and then increased again. For CuI-doped BTSS_0.2, the *κ* value decreased with increasing temperature. The *κ* values of both doped samples were larger than those of the un-doped samples due to increased carrier concentration and mobility near room temperature.

When the *κ*(*T*) values of the compounds were compared with other reported examples (dashed lines),^4,9^ the Cu-doped Bi_2_Te_2.3_Se_0.3_S_0.4_ compounds exhibited the lowest thermal conductivity value (0.82 W m^−1^ K^−1^ at 300 K). The relatively high thermal conductivity of CuI-doped BTSS (*x* = 0.05 and 0.2) compounds was due to the high electrical conductivity.

Fig. 6(b) shows the lattice thermal conductivity of the samples. The *κ*_L_ value of the CuI-doped BTSS_0.2 was lower than that of the doped BTSS_0.05 sample owing to the increased disorder, which suppressed the bipolar effect compared with doped BTSS_0.05. The *κ* values of the un-doped samples (BTSS_0.05 and 0.2) increased due to the bipolar diffusion effect with increasing temperature. As mentioned above, we believe that the bipolar effect occurs readily due to holes in the anti-site defect impurity band owing to electron localization near the band tail. Ironically, despite the BTSS_0.2 sample including a large amount of disorders compared with the BTSS_0.05 sample, the *κ* value of the BTSS_0.2 sample was larger than that of BTSS_0.05 near room temperature.

Recently, Thesberg *et al.*^36^ reported that the theoretically calculated Lorenz number can be significantly increased between the valence and conduction bands in the bipolar system, with a value several orders of magnitude higher than the Lorentz number of a conventional metallic system.^36^ In our case, this phenomenon can also be applied, because the chemical potential located at the conduction band tail is relatively different to that at the centre of the band gap, compared with degenerated CuI-doped samples. Therefore, we speculated on the thermal conductivity and Lorentz number within a single parabolic band assumption. As a result, we confirmed that the chemical potential of un-doped samples was situated in the localization region and that the bipolar effects for the CuI-doped samples in the *κ* value were suppressed by the chemical potential tuning up at high temperature compared with un-doped samples.

**Fig. 7:**
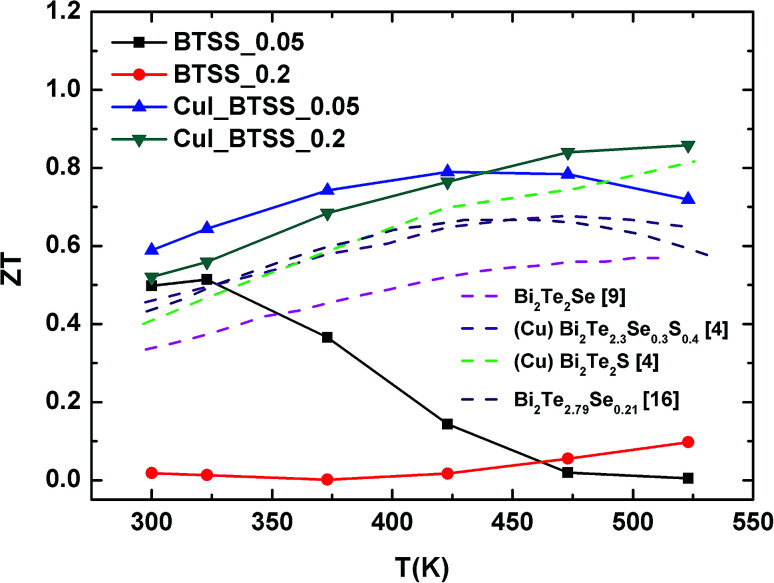
Temperature-dependent *ZT* values for (CuI)_*y*_(Bi_2_Te_3_)_0.95−*x*_(Bi_2_Se_3_)_*x*_(Bi_2_S_3_)_0.05_ (*x* = 0.05, 0.2; *y* = 0.0, 0.003) bulk compounds. Pink, purple, green, and blue dashed lines represent *ZT* values for Bi_2_Te_2_Se,^9^ Cu-doped Bi_2_Te_2.3_Se_0.3_S_0.4_,^4^ Cu-doped Bi_2_Te_2_S,^4^ and Bi_2_Te_2.79_Se_0.21_,^16^ respectively, from other references.

The temperature-dependent *ZT* values for all samples are presented in Fig. 7. The *ZT* values for CuI-doped samples were enhanced in comparison with the un-doped samples BTSS_0.05 and BTSS_0.2, and compared with previously reported examples. The highest *ZT* value was 0.86 at 523 K for CuI-doped BTSS_0.2. Especially, the *ZT* value of the CuI-doped BTSS_0.2 sample was significantly increased because the chemical potential located in the localized region moved to the conduction band or delocalized region. Although the BTSS_0.2 sample included a large amount of disorders compared with BTSS_0.05, the *ZT* value of the BTSS_0.2 sample was drastically increased.

## 4. Conclusions

In summary, we have identified a new approach to chemical potential tuning through CuI doping and disorder effects caused by variations in the Se and S concentrations in (CuI)_*y*_(Bi_2_Te_3_)_0.95−*x*_(Bi_2_Se_3_)_*x*_(Bi_2_S_3_)_0.05_ (*x* = 0.05, 0.2; *y* = 0.0, 0.003) compounds. Disorders induced by lattice strain in the matrix were confirmed by XRD peak broadening and HR-TEM images. Furthermore, variable range hopping transport of carriers was observed from the electrical resistivity at low temperature. We believe that disorders created a disorder potential within the matrix and formed a band tail, resulting in a sharp density of states. Therefore, when the chemical potential was located near the band tail, the carriers can be strongly localized by the disorder potential.

To improve the thermoelectric properties, the chemical potentials were tuned to the delocalized region by Cu/I co-doping, even in the presence of disorders. From chemical potential tuning in the disordered system, thermoelectric properties were significantly improved in terms of charge localization and delocalized transition. The maximum *ZT* value of about 0.86 was reached at 523 K, which was a relatively high *ZT* value for n-type thermoelectric materials among previously reported examples. We demonstrated that the thermoelectric properties can be enhanced in the disordered system by converting the localized state into a delocalization state through chemical potential tuning.

## Conflicts of interest

There are no conflicts to declare.

## Supplementary Material
